# Specific relations of dimensional anxiety and manifest anxiety disorders during pregnancy with difficult early infant temperament: a longitudinal cohort study

**DOI:** 10.1007/s00737-019-01015-w

**Published:** 2020-01-11

**Authors:** Freya Thiel, Laura Iffland, Filip Drozd, Silje Marie Haga, Julia Martini, Kerstin Weidner, Malin Eberhard-Gran, Susan Garthus-Niegel

**Affiliations:** 1 Department of Psychotherapy and Psychosomatic Medicine, Faculty of Medicine, Carl Gustav Carus University Hospital, Technische Universität Dresden, Fetscherstraße 74, 01307 Dresden, Germany; 2 Institute for Clinical Psychology and Psychotherapy, Technische Universität Dresden, Chemnitzer Straße 46, 01187 Dresden, Germany; 3 Department for Infant Mental Health, Regional Centre for Child and Adolescent Mental Health (RBUP) Eastern and Southern Norway, Gullhaugveien 1-3, 0484 Oslo, Norway; 4 Department of Psychiatry and Psychotherapy, Faculty of Medicine, Carl Gustav Carus University Hospital, Technische Universität Dresden, Fetscherstraße 74, 01307 Dresden, Germany; 5 HØKH, Research Centre, Akershus University Hospital, 1478 Lørenskog, Norway; 6 Institute of Clinical Medicine, Campus Ahus, University of Oslo, 1478 Lørenskog, Norway; 7 Department of Child Health and Development, Norwegian Institute of Public Health, 0213 Oslo, Norway

**Keywords:** Prenatal anxiety, Fear of childbirth, Infant temperament

## Abstract

Anxiety in the antenatal period is a common experience, associated with adverse consequences for mother and child. Specific types of prenatal anxiety may have unique associations with infant temperament. This study examines the prospective relationships between general prenatal anxiety, fear of childbirth, and specific prenatal anxiety disorders and early infant temperament 8 weeks postpartum. Data were derived from the Akershus Birth Cohort (ABC), a longitudinal cohort study which targeted all women scheduled to give birth at Akershus University Hospital, Norway. Psychometric measures pertained to general prenatal anxiety (Hopkins Symptom Checklist), fear of childbirth (Wijma delivery expectancy questionnaire), screening for manifest prenatal anxiety disorders based on questions from the mini-international neuropsychiatric interview, and difficult infant temperament (Infant Characteristics Questionnaire). The sample for the present study included 2206 women. General prenatal anxiety, fear of childbirth, agoraphobia, generalized anxiety disorder, and specific phobia presented unique significant prospective contributions to difficult infant temperament 8 weeks postpartum. Separate hierarchical regression models indicated that general prenatal anxiety and fear of childbirth provided the strongest unique contributions. Considering the burden on mothers and the potential long-term effects on child development, the findings of this study highlight the importance of screening women for different types of prenatal anxiety in routine obstetric care. Clinical awareness of the condition and its consequences is warranted. Due to the complexity of infant temperament as a construct with various influences, future research should consider mechanisms and influential factors pertaining to the relationship between prenatal anxiety and infant temperament.

In the past, pregnancy has widely been viewed as a low-risk period for mental disorders and even as a protective factor against mental health issues (Elliott et al. 1983). However, prior research shows that anxiety symptoms are common and may even be more prevalent toward the end of pregnancy than postpartum (e.g., Goodman et al. 2014). Pregnancy may affect specific types of anxiety disorders in differential ways. For instance, while obsessive-compulsive disorder may be triggered or worsened during pregnancy (Abramowitz et al. 2003), panic disorder may ameliorate (George et al. 1987). For some women, fear of childbirth itself may develop above the threshold of diagnostic standards for a specific phobia (Hofberg and Brockington 2000).

With approximately 20% of pregnant women experiencing fear of childbirth (Areskog et al. 1981; Jolly et al. 1999; Rouhe et al. 2009), it presents an important women’s health issue with adverse consequences for the infant. In most cases, it includes fear for the child’s health and well-being, fear of pain, death or physical injury, or loss of control and may reach its peak in late pregnancy, when childbirth is actually approaching (Parker 1986; Sjögren 1997; Szeverényi et al. 1998; Lowe 2000; Geissbuehler and Eberhard 2002; Melender 2002; Saisto and Halmesmäki 2003; Preis et al. 2018). Severe fear of childbirth has been linked to primiparity, previous negative birth experience(s), previous emergency C-section, smoking, lack of support, general anxiety, low self-esteem, depression, and relationship dissatisfaction (e.g., Saisto and Halmesmäki 2003; Fenwick et al. 2009; Haines et al. 2011; Størksen et al. 2013; Lukasse et al. 2014). Childbirth-related anxiety is associated with an increased number of planned cesarean sections (Størksen et al. 2015) which in turn put mother and child at an increased risk for medical complications. Similarly, anxiety during pregnancy is associated with adverse outcomes, such as premature birth, longer labor (Parker 1986), newborn asphyxia (Herrera et al. 1992), breastfeeding difficulties, postpartum depression (Eberhard-Gran et al. 2002), and implications for the mother–infant relationship (Areskog et al. 1984). Nonetheless, investigations into the relationship between fear of childbirth and general anxiety symptoms during pregnancy as well as their potential impact on infant temperament remain scarce.

Accumulating evidence suggests a persistent impact of maternal stress during pregnancy on infant development. Infants and toddlers exposed to maternal distress during pregnancy may be at risk for fearful behavior and difficult temperament (e.g., Huizink et al. 2002; Bergman et al. 2007). Temperamental traits are thought to manifest early in life and are relatively stable over contexts and time as early as a few weeks following birth (Worobey and Blajda 1989; Austin et al. 2005; Shiner et al. 2012; Bornstein et al. 2015). Difficult infant temperament may include high reactivity, proneness to negative emotional expressions, and low emotional flexibility and emotion regulation (Chess and Thomas 1989; Rothbart and Bates 2006; McCrory et al. 2012; Abulizi et al. 2017). Adversities in early temperament and emotion regulation may in turn reinforce behavioral difficulties during childhood, creating long-lasting adverse effects (e.g., O’Connor et al. 2002; Polte et al. 2019). While positive postpartum influences may act as moderators, the relationship of antenatal distress and child outcomes persists even after controlling for postpartum factors such as maternal postpartum mental health (Bergman et al. 2008). There is thus a crucial need to investigate the role of anxiety during pregnancy in order to develop appropriate preventive interventions.

Our previous research demonstrates the need to distinguish between fear of childbirth and anxiety during pregnancy, documenting prevalence rates of 6–8% for fear of childbirth and around 9% for anxiety (Storksen et al. 2012). Presence of anxiety significantly increased the odds of also reporting fear of childbirth. However, more than half the women with fear of childbirth did not report anxiety (Storksen et al. 2012). These findings are further in line with previous work highlighting general anxiety as a risk factor for fear of childbirth (Saisto and Halmesmäki 2003; Fenwick et al. 2009). While fear of childbirth may in extreme cases reach the diagnostic threshold for specific phobia (i.e., tokophobia), it is a common experience and as opposed to pathological anxiety, may follow a normal distribution (Parker 1986; Saisto and Halmesmäki 2003).

Nonetheless, the current literature regarding different associations of anxiety versus fear of childbirth and child outcomes remains inconclusive. In a recent systematic review including 32 studies (Korja et al. 2017), four out of seven studies reported associations between fear of childbirth and infant attention (e.g., Huizink et al. 2003), emotion regulation (e.g., Henrichs et al. 2009), and negative reactivity (e.g., Blair et al. 2011), while three studies did not (e.g., Baibazarova et al. 2013). Similarly, while 14 studies reported links between anxiety and children’s negative reactivity (e.g., McMahon et al. 2013), eight studies reported no association (e.g., Blair et al. 2011). These inconclusive findings may be explained by methodological variations (e.g., assessment tools, assessment times, sample sizes). While a predominance of studies documents a relationship between prenatal anxiety and infant temperament, the relationship between fear of childbirth and temperament is less supported.

Using longitudinal data from a large sample of pregnant Norwegian women, this study examines the prospective relationships between general (dimensional) prenatal anxiety, fear of childbirth, as well as specific (manifest) anxiety disorders during pregnancy and difficult early infant temperament at 8 weeks postpartum, aiming to assess their unique contributions to difficult infant temperament.

## Methods

### Design and participants

Data were derived from the Akershus Birth Cohort (ABC), a prospective cohort study which targeted all women scheduled to give birth at Akershus University Hospital, Norway. Recruitment took place from November 2008 to April 2010. Expectant mothers were recruited during their routine fetal ultrasound examination around gestational week 17. Of the eligible women (able to complete a questionnaire in Norwegian), 80% (*n* = 3752) agreed to participate and returned the first questionnaire.

**Fig. 1:**
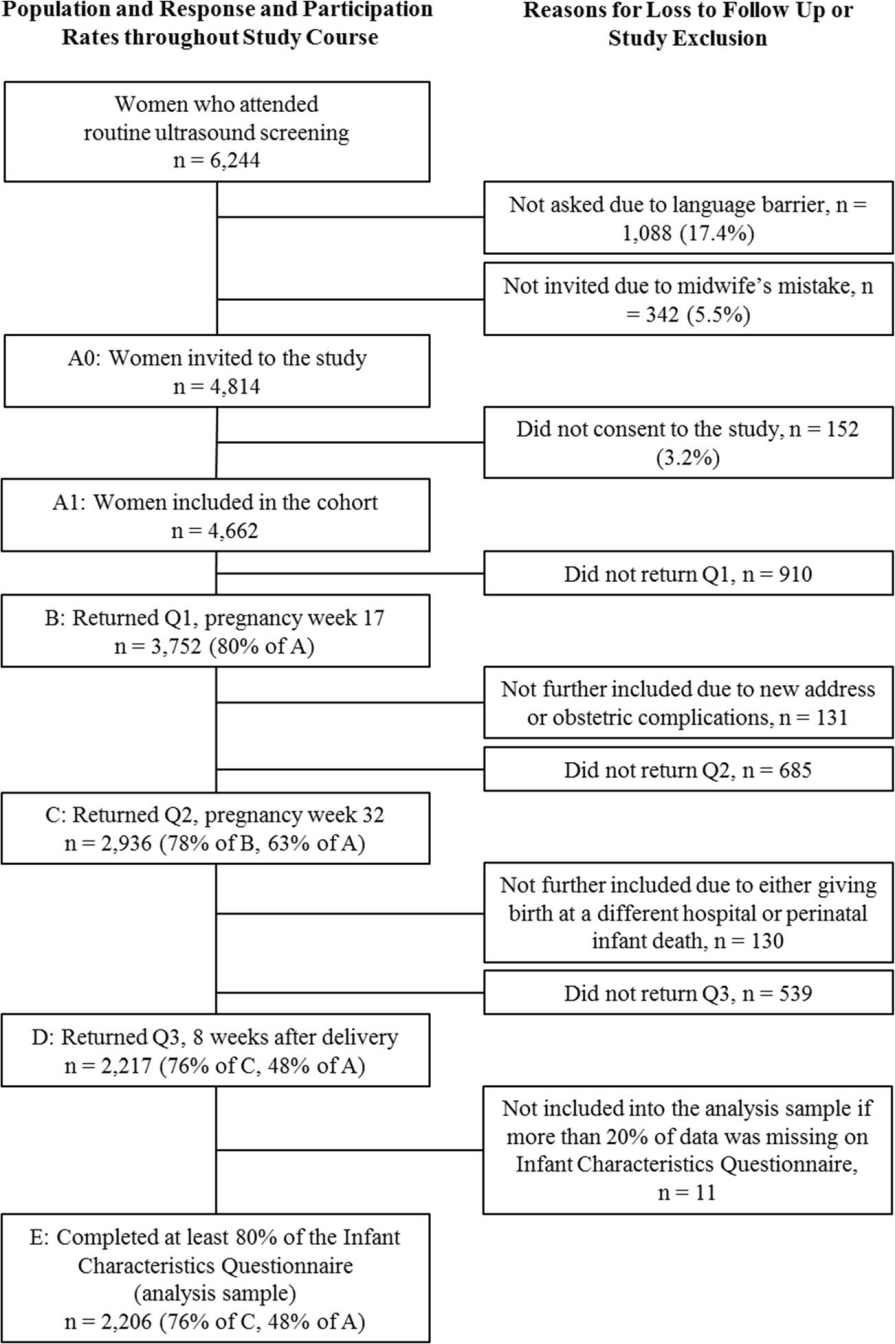
Study flow chart

Further self-report assessments took place at pregnancy week 32 (T2) and 8 weeks postpartum (T3). Eligibility rates decreased slightly at T2 and T3 as a result of women moving away or being withdrawn from the study due to severe obstetric complications. For the current study, we utilized hospital birth record information (i.e., sociodemographic and medical information) and self-report data from all three assessment points. We included women who completed the Infant Characteristics Questionnaire (ICQ) at T3, yielding a sample of *n* = 2206 (see Fig. 1).

The ABC study received ethical approval from the Regional Committees for Medical and Health Research Ethics in Norway (approval number S-08013a) and was conducted in accordance with the Declaration of Helsinki.

### Measures

#### Prenatal anxiety

We assessed general prenatal anxiety symptoms and manifest anxiety disorders during gestational weeks 17 and 32. Prenatal anxiety symptoms were measured using the Norwegian versions of the anxiety scale (SCL-anxiety [SCL-A]) of the Hopkins Symptom Checklist (SCL-25). Anxiety disorder measurements were based on questions from the mini-international neuropsychiatric interview (MINI).

The SCL-A comprises 10 items measuring dimensional general anxiety symptoms during the previous week (Winokur et al. 1984), scored from 1 (“not at all”) to 4 (“extremely”), yielding total scores between 10 and 40 (Nettelbladt et al. 1993). In accordance with previous studies, we defined presence of anxiety as SCL-A ≥ 18 (e.g., Eberhard-Gran et al. 2003; Storksen et al. 2012). Reliability in the current study was *α* = 0.75 at gestational week 17 and *α* = 0.78 at gestational week 32.

An extensive battery of self-administered questions regarding anxiety disorders from the MINI was created for use in the current study and administered in gestational weeks 17 and 32. The MINI is a short, structured clinical interview designed for use in epidemiological studies and clinical trials; it has proven good psychometric properties (Lecrubier et al. 1997; Sheehan et al. 1997) and enables researchers to diagnose psychiatric disorders according to DSM-IV-TR or ICD-10 categories (Sheehan et al. 1998). The participants’ answers were coded according to DSM-IV-TR diagnostic criteria for the following anxiety categories: panic disorder (e.g., “Have you had spells or attacks when you suddenly felt fear or panic?”), agoraphobia (e.g., “Do you feel anxious in places or situations where escaping is difficult? (for example, in a large crowd, in a queue, or away from home alone)”), specific phobia (e.g., “Do any of the following items or situations trigger fear or panic in you?” – e.g., flights, heights etc.), social phobia (e.g., “In the past month, were you fearful or embarrassed being watched, being the focus of attention, or fearful of being humiliated? (e.g., speaking in public or being in social situations))”, obsessive-compulsive disorder (OCD, e.g., “In the past month, did you do something repeatedly without being able to resist doing it? (e.g., cleaning or washing, counting or checking things over and over”)), posttraumatic stress disorder (PTSD, e.g., “Have you ever experienced or been involved in a dramatic and terrifying event? (e.g., accident, violence/abuse against yourself or others) – no; yes, and I reacted with intense fear, helplessness or horror; yes, but I did not let it get to me”), and general anxiety disorders (GAD, e.g., “Have you at times been anxious almost daily, without the concern being associated with particular situations?”) (Garthus-Niegel et al. 2013; Osnes et al. 2019). Agoraphobia and panic disorder were coded as two separate categories. The time between the first and second questionnaire was only 4 months. In order to avoid overlapping of reported symptoms between the two questionnaires, the GAD duration criterion was relaxed to 1 month. We measured the prevalence of the MINI-anxiety categories at gestational weeks 17 and 32, although OCD and PTSD were assessed only at week 17 (Osnes et al. 2019).

We computed a continuous variable representing general prenatal anxiety by averaging T1 and T2 SCL-A scores and dichotomous variables representing distinct prenatal anxiety disorder symptoms for Mini-anxiety categories administered at both T1 and T2. We coded “0” if Mini-anxiety categories were not indicated at T1 or T2, and “1” if they were indicated at T1 and/or T2. For descriptive reasons, we also established a dichotomous variable representing the occurrence of prenatal anxiety, coded as “0” if SCL-A scores at both T1 and T2 < 18 and coded as “1” if SCL-A scores at T1 and/or T2 ≥ 18.

#### Fear of childbirth

We measured fear of childbirth during gestational week 32 (T2) using a Norwegian version of the Wijma delivery expectancy/experience questionnaire version A (W-DEQ) (Garthus-Niegel et al. 2011). The W-DEQ comprises 33 items asking expectant mothers to rate their expectations of the upcoming childbirth from 0 (“not at all”) to 5 (“extremely”) using different adjectives and nouns (e.g., “weak,” “safe,” “tense,” “desolate,” “hopelessness,” “self-confidence,” “trust,” “panic”), yielding total scores between 0 and 165. In accordance with previous studies, we defined fear of childbirth as W-DEQ ≥ 85 (Ryding et al. 1998). Reliability in the current study was *α* = 0.92.

#### Infant temperament

Early infant temperament was assessed at 8 weeks postpartum, using a 10-item adapted version of the “fussy/difficult” subscale of the Infant Characteristics Questionnaire (ICQ). Mothers rated their infants’ usual mood and temperament (e.g., “Your child is usually easy to pacify when he/she is crying”; “My child is so demanding that he/she would pose a major problem for most parents”) from 1 (“completely disagree”) to 7 (“completely agree”) with higher scores indicating greater reported infant difficultness. The “fussy/difficult” subscale of the ICQ has good psychometric properties (Bates et al. 1979) and reliability of the 10-item adapted subscale was *α* = 0.82.

#### Sociodemographic and childbirth-related information

Included hospital birth record information pertained to education, employment and marital status, smoking, alcohol consumption, and use of antidepressants or anti-anxiety medication during pregnancy, maternal age at delivery, parity, premature birth, mode of delivery, child sex, and obstetric complications (e.g., uterine rupture, eclampsia, infections). Severe infant health complications at birth (i.e., admission to the Neonatal Intensive Care Unit) were reported by the mother at 8 weeks postpartum.

### Statistical analysis

Analyses were performed in SPSS Statistics version 25. We used mean imputation if a participant completed at least 80% of a scale. First, we investigated potential differences regarding sociodemographic and childbirth-related information between those with and without prenatal anxiety (dichotomous variable) using *t* and chi-square tests. Second, we used bivariate Pearson correlations to examine associations between ICQ scores and the averaged SCL-A variable and Mini-anxiety categories, W-DEQ, as well as sociodemographic and childbirth-related information. Third, we ran separate hierarchical regression models for Mini-anxiety subscales, SCL-A, and W-DEQ scores. In each model, we controlled for sociodemographic and childbirth-related factors predicting difficult infant temperament in the first step and added the separate anxiety measure in the second step.

## Results

### Demographics and descriptive statistics

Participants were between 19 and 46 years old (*M* = 31.33, *SD* = 4.62). Most were married or living with their partner (96%), employed on a full-time basis (83%), and had at least 12 years of education (65%). Only very few women indicated having smoked (1% “daily,” 2% “now and then”), consumed alcohol (0.5%), or having used antidepressant or anti-anxiety medication (1%) during pregnancy. Half of the sample (50%) was primiparous, and most delivered vaginally (85%) and at term (93%; i.e., delivery within 21 days prior to ultrasound calculated date or at least 258 days after last menstruation). While around a third reported at least one obstetric complication (34%), only few women indicated severe infant health issues at birth (6%). Descriptive statistics for SCL-A, W-DEQ, Mini-anxiety, and ICQ can be found in Table 1.

**Table 1:** Descriptive statistics for difficult infant temperament and study predictors

	Measure range	Min–max	M (SD)	*n* (%)
Pregnancy week 17				
SCL-A	10–40	10–36	12.88 (2.95)	169 (7.8)
Panic disorder	Dichotomous			11 (0.5)
Agoraphobia	Dichotomous			89 (4.1)
GAD	Dichotomous			23 (1.0)
Specific phobia	Dichotomous			71 (3.2)
Social phobia	Dichotomous			40 (1.8)
OCD	Dichotomous			53 (2.4)
PTSD	Dichotomous			4 (0.2)
Any anxiety disorder	Dichotomous			215 (9.8)
Pregnancy week 32				
SCL-A	10–40	10–32	12.77 (3.09)	163 (8.3)
W-DEQ	0–165	2–145	56.97 (19.80)	153 (8.0)
Panic disorder	Dichotomous			15 (0.8)
Agoraphobia	Dichotomous			69 (3.6)
GAD	Dichotomous			37 (1.9)
Specific phobia	Dichotomous			70 (3.5)
Social phobia	Dichotomous			34 (1.7)
Any anxiety disorder	Dichotomous			162 (8.4)
8 weeks following delivery				
ICQ	10–70	10–64	25.67 (9.04)	

*SCL-A*, anxiety scale of the Hopkins Symptom Checklist (SCL-25). Panic disorder, agoraphobia, GAD, specific phobia, social phobia, OCD, PTSD measured with self-administered items from the mini-international neuropsychiatric interview (Mini-anxiety). *Any anxiety disorder*, at least one of the Mini-anxiety disorders. *OCD*, obsessive compulsive disorder; *GAD*, generalized anxiety disorder; *W-DEQ*, Wijma delivery expectancy/experience questionnaire version A; *ICQ*, infant characteristics questionnaire. For continuous variables, *n* (%) indicates scoring above measure cutoff, i.e., SCL-A ≥ 18 and W-DEQ ≥ 85

Women with anxiety symptoms during pregnancy (i.e., SCL-A ≥ 18 at T1 and/or T2) were significantly younger (*M* = 29.31, *SD* = 5.20) than those without anxiety (*M* = 31.60, *SD* = 4.47), *t*(304.34) = 6.70, *p* < .001, and were more likely to have had less than 12 years of education (anxiety 50.0%, no anxiety 70.0%, *X*^2^(2,*N* = 2107) = 38.59, *p* < 0.001), be unmarried or separated (anxiety 4.7%, no anxiety 2.1%, *X*^2^(2,*N* = 2166) = 8.52, *p* = .01), and unemployed (anxiety 14.8%, no anxiety 4.7%, *X*^2^(4,*N* = 2073) = 44.35, *p* < .001).

### Antenatal anxiety and difficult infant temperament

ICQ scores were associated with SCL-A, W-DEQ, agoraphobia, GAD, specific phobia, and social phobia, as well as younger maternal age, lower employment status, primiparity, more stressful modes of delivery such as assisted vaginal deliveries and unscheduled cesarean sections, obstetric complications, and male child sex (see Table 2).

**Table 2:** Pearson correlation coefficients for difficult infant temperament and study predictors, demographics, and childbirth-related information

	Difficult temperament	Prenatal anxiety	Fear of childbirth	Panic disorder	Agora-phobia	GAD	Specific phobia	Social phobia	OCD	PTSD
Study outcome										
Difficult infant temperament	–	0.16**	0.21**	0.02	0.10**	0.07*	0.05*	0.05*	0.03	0.02
Study predictors										
General prenatal anxiety		–	0.32**	0.23**	0.29**	0.33**	0.23**	0.21**	0.20**	0.12**
Fear of childbirth			–	0.07*	0.14**	0.08**	0.12**	0.13**	0.07**	0.03
Panic disorder				–	0.19**	0.24**	0.14**	0.09**	0.10**	0.10**
Agoraphobia					–	0.17**	0.24**	0.27**	0.10**	0.08**
GAD						–	0.21**	0.19**	0.18**	0.06**
Specific phobia							–	0.35**	0.14**	0.04
Social phobia								–	0.10**	0.06**
OCD									–	0.06**
PTSD										–
Demographics										
Marriage status	− 0.004	− 0.06**	− 0.05*	0.02	− 0.04	0.03	0.01	0.01	0.004	0.01
Employment status	0.04*	0.16**	0.03	0.06*	0.17**	0.09**	0.08**	0.07**	0.05*	0.12**
Education	0.02	− 0.17**	0.002	− 0.03	− 0.12**	− 0.04	− 0.04	− 0.03	− 0.01	− 0.01
Maternal age	− 0.07*	− 0.18**	− 0.01	− 0.04	− 0.06*	− 0.05*	− 0.08**	− 0.09**	− 0.09**	− 0.02
Smoking during pregnancy	0.01	0.13**	0.03	0.05*	0.08**	0.01	0.06**	0.04	0.01	0.09*
Alcohol during pregnancy	− 0.003	− 0.003	− 0.01	− 0.01	0.04	− 0.01	0.01	0.03	− 0.01	− 0.003
Antidepressant/anti-anxiety med.	0.01	0.12**	0.04	− 0.01	0.12*	0.08*	0.07*	0.01	0.05*	− 0.004
Childbirth-related										
Primiparity	0.12**	0.05*	0.14**	− 0.01	0.05*	0.01	0.01	0.06**	− 0.01	0.00
Prematurity	0.03	0.01	0.03	0.01	0.03	0.02	0.004	− 0.02	0.01	0.09**
Child sex	0.10*	0.01	0.01	− 0.02	0.01	− 0.02	− 0.01	0.01	0.01	− 0.04*
Delivery mode	0.08*	0.01	0.08**	0.01	0.02	− .001	0.03	0.03	− 0.01	0.01
Obstetric complications	0.09**	− .01	0.05*	0.003	0.01	− 0.01	− 0.003	0.004	− 0.01	− 0.01
Infant health complications	0.03	0.01	0.04	− 0.03	− 0.01	0.02	0.0	0.03	− 0.02	− 0.01
Breastfeeding	0.01	0.11**	0.08**	0.01	0.03	0.01	0.10	0.01	− 0.03	0.001
Prenatal attachment	0.01	0.13**	− 0.06**	0.03	0.02	0.05*	0.01	0.02	0.01	− 0.004

Difficult infant temperament measured with infant characteristics questionnaire. General prenatal anxiety measured with SCL-anxiety (SCL-A) of the Hopkins Symptom Checklist (SCL-25). Fear of childbirth measured with Wijma delivery expectancy/experience questionnaire version A (W-DEQ). *GAD*, generalized anxiety disorder; *OCD*, obsessive compulsive disorder; *PTSD*, posttraumatic stress disorder. Panic disorder, agoraphobia, GAD, specific phobia, social phobia, OCD, PTSD measured with self-administered items from the mini-international neuropsychiatric interview (Mini-anxiety). Delivery mode coded (0) vaginal, (1) elective cesarean section, (2) assisted vaginal, (3) emergency cesarean section

Agoraphobia, GAD, and specific phobia contributed to the prediction of difficult infant temperament when controlling for employment status, maternal age, parity, child sex, and obstetric complications. In all models, primiparity and giving birth to a male child contributed significantly, explaining 3% of the variance in difficult infant temperament. Agoraphobia, GAD, and specific phobia added 0.7%, 0.4%, and 0.2% of the explained variance, respectively (see Table 3).

**Table 3:** Separate hierarchical regression models for difficult infant temperament by maternal antenatal anxiety

Variable	Model 1: agoraphobia	Model 2: GAD	Model 3: specific phobia	Model 4: social phobia	Model 5: prenatal anxiety	Model 6: fear of childbirth
Block 1:						
Control variables						
Employment	0.03	0.04	0.04	0.04	0.02	0.03
Maternal age	− 0.03	− 0.03	− 0.03	− 0.03	− 0.003	− 0.03
Primiparity	0.09*	0.09*	0.09*	0.09*	0.09*	0.06*
Child sex male	0.10*	0.10*	0.10*	0.10*	0.10*	0.11*
Delivery mode	0.04	0.04	0.03	0.04	0.03	0.03
Obstetric compl.	0.03	0.03	0.03	0.03	0.04	0.03
Block 2:						
Agoraphobia	0.08*					
GAD		0.07*				
Specific phobia			0.05*			
Social phobia				0.03		
General prenatal anxiety					0.15*	
Fear of childbirth						0.20*
R^2^	0.04*	0.04*	0.03*	0.03*	0.05*	0.0*
R^2^ change	0.01*	0.004*	0.002*	0.001	0.02*	0.04*

Values represent standardized regression coefficients (Beta). Difficult infant temperament measured with infant characteristics questionnaire. *GAD*, generalized anxiety disorder. Agoraphobia, GAD, specific phobia, social phobia measured with self-administered items from the ,mini-international neuropsychiatric interview (Mini-anxiety). General prenatal anxiety indicated by scores on the SCL-anxiety (SCL-A) of the Hopkins Symptom Checklist (SCL-25). Fear of childbirth indicated by scores on the Wijma delivery expectancy/experience questionnaire version A (W-DEQ)

*p < 0.05

Hierarchical regression models including SCL-A and W-DEQ explained more variance in difficult infant temperament than the manifest anxiety disorders. SCL-A scores added 2.3% of explained variance in infant temperament, resulting in the overall model explaining 5.3%, *F*(1.2065) = 49.13, *p* < 0.001. Similarly, W-DEQ scores added 3.9% of explained variance, yielding an overall model explaining 7.1% of variance in difficult infant temperament, *F*(1.1834) = 76.15, *p* < .001.

## Discussion

We examined the prospective relationships between general prenatal anxiety, manifest anxiety disorders during pregnancy, as well as fear of childbirth and difficult early infant temperament at 8 weeks postpartum in a sample of 2206 Norwegian women, aiming to assess the unique contributions to difficult infant temperament as rated by the mother. Our main findings pertained to unique contributions of general prenatal anxiety, fear of childbirth, agoraphobia, GAD, and specific phobia to difficult infant temperament, with the strongest unique contributions for general prenatal anxiety and fear of childbirth. While previous research has established the association between prenatal anxiety and maternal perinatal distress and difficult infant temperament (e.g., Huizink et al. 2002; Davis et al. 2004; Davis et al. 2007), our findings add to the literature by documenting associations between different types of prenatal anxiety and difficult infant temperament.

Importantly, our findings highlight the potential consequences of fear of childbirth on the infant. Fear of childbirth is a common experience and, as opposed to psychopathological anxiety, may follow a normal distribution (Parker 1986; Saisto and Halmesmäki 2003). Our finding of fear of childbirth predicting difficult early infant temperament thus presents important clinical implications. Women presenting levels of fear of childbirth not reaching pathological dimensions may benefit from additional support. Further, education of clinical staff to raise awareness of the potential influences of fear of childbirth on the infant and challenges in the transition to parenthood are warranted.

The prospective association between prenatal anxiety and infant temperament documented herein may be explained using the developmental model of fetal programming, according to which prenatal exposure can prompt long-term developmental responses in the organism, affecting neurobiology and behavior (Egliston et al. 2007; Glover 2011; McCrory et al. 2012). Increased maternal stress hormones, produced by the mother’s hypothalamic-pituitary-adrenal (HPA) axis, may impact fetal development of structural and functional neural systems, affecting emotional and behavioral responses in infancy (Egliston et al. 2007; McCrory et al. 2012). This process may be mediated by placental functioning as stress-related downregulation of the placental barrier enzyme 11β-HSD2 may increase fetal exposure to maternal cortisol (O’Donnell et al. 2012; Blakeley et al. 2013). While the exact mechanism behind the effects of different types of prenatal anxiety on fetal neurobiological development remains unclear, maternal stress during pregnancy has been associated with altered key structures for social processing and emotional self-regulation in the infant, for instance, the amygdala and the prefrontal and lateral temporal cortices (Sandman et al. 2011; Buss et al. 2012).

Our findings are of particular importance due to potential long-term consequences for the child. Negative reactivity and behavioral inhibition, which can both be conceptualized as parts of childhood temperament, have been shown to be linked to later mental health adversities, such as depression or anxiety disorder (e.g., Hirshfeld-Becker et al. 2007; Hudson et al. 2011; Sayal et al. 2014). Further, as part of negative reactivity, persistent excessive infant crying has been linked to difficulties in emotional self-regulation, attention regulation, and other social behavioral aspects (e.g., Desantis et al. 2004; Korja et al. 2014; Martini et al. 2017). Additionally, specific areas of the prefrontal cortex and limbic structures may play a crucial role in the link between temperament and later psychopathology (Whittle et al. 2006), as noted above, areas which may present alterations linked to maternal prenatal stress.

Nonetheless, additional environmental and biological factors may affect the association between prenatal anxiety and child outcomes. For instance, although we did not find an association between maternal alcohol consumption or smoking during pregnancy and infant temperament, these behaviors are related to decreased mental health, may pose further risk factors for altered fetal development (Kaplan-Estrin et al. 1999), and may moderate or mediate the relationship between maternal prenatal anxiety and infant temperament (Korja et al. 2017). Moreover, infant temperament may have genetic (Braungart et al. 1992) and epigenetic components (Gartstein and Skinner 2018). Additionally, factors related to early parental attachment and caregiving could further provoke or protect against early temperamental difficulties, and later behavioral inhibition and emotional reactivity (e.g., Nachmias et al. 1996; Bergman et al. 2008; Grant et al. 2010). It is important to note that there may be cross-cultural differences in the development of temperamental traits (Gartstein et al. 2006). While most prior research has utilized Western samples, culture-bound family structures, views on child care, and support during pregnancy and the transition into motherhood may affect the link between maternal prenatal distress and infant temperament. To this end, a recent investigation of prenatal distress and infant temperament in an Indian sample has found no association (Bhat et al. 2015), highlighting the need for future research on cultural differences pertaining to the link between prenatal anxiety and infant temperament.

## Limitations

Data on anxiety, fear of childbirth, and infant temperament were based on self-report. The women’s clinical records did not provide information related to anxiety. Nonetheless, previous research has reported associations between self-reported maternal prenatal depression and infant temperament (e.g., Glover 2011). Maternal self-reports of prenatal anxiety may be associated with maternal self-reported prenatal fetal temperament suggesting a reporting bias (Mebert 1991). However, moderate agreement between maternal and paternal ratings of infant temperament has been documented (Austin et al. 2005). Similarly, it should be noted that we could not establish the contribution of concurrent (postpartum) anxiety to maternal ratings of infant temperament. Future studies should take current psychological states into account in order to examine potential mood biases in maternal ratings of the infant. Further, there is currently no gold standard instrument to assess prenatal anxiety (Meades and Ayers 2011), and heterogeneous use of instruments, cutoff scores, and time points make inter-study comparisons rather difficult (Field 2018). While the significance and relevance of the subscale used here has repeatedly been shown (e.g., O’Donnell et al. 2012), it should be noted that there are gold standard observational measures for infant behavior and temperament which should be used in future studies to replicate the findings presented herein. Another limitation to consider is potential comorbidity among anxiety disorders. Here, most Mini-anxiety categories were significantly associated with one another, indicating some degree of comorbidity. Coefficients were low, except the association between social and specific phobia which was on the lower end of the moderate range (Table 2). We therefore conclude that there is evidence for an association, but not for high comorbidity.

Moreover, our sample was relatively homogeneous, being based on Norwegian-speaking women, with the majority being Caucasian. Other ethnic groups may diverge regarding risk factors and prenatal anxiety (Rubertsson et al. 2014; Liu et al. 2016). Study participation was associated with a slight social gradient (Garthus-Niegel et al. 2014, 2015) and prior attrition analyses indicated somewhat selective attrition over the study course further limiting generalizability of our findings (Garthus-Niegel et al. 2018). For instance, attrition analyses revealed that women with severe mental distress during pregnancy may have been more likely to drop out of the study, as indicated by lower response rates at 32 weeks by women with high EPDS scores at 17 weeks. Nonetheless, it should be noted that results are not necessarily influenced by selection bias when examining associations (Nilsen et al. 2009). Additionally, while the current sample is fairly psychologically healthy and though effect sizes were rather small, we still find a link between maternal prenatal anxiety and early infant temperament with important clinical implications.

## Conclusion

Our study reveals unique prospective contributions of general prenatal anxiety, manifest anxiety disorders, and fear of childbirth to difficult early infant temperament at 8 weeks postpartum. Considering the burden on mothers posed by prenatal anxiety and fear of childbirth, as well as potentially detrimental long-term effects on neurobiological and socio-behavioral child development, our findings highlight the importance of screening women for different types of anxiety during pregnancy. To date, routine prenatal check-ups typically focus on medical and somatic aspects, possibly neglecting maternal psychological states. As maternal anxiety during pregnancy is a common experience with potentially transgenerational effects, clinical awareness of the condition and its consequences is warranted in order to intervene effectively. For instance, expectant mothers suffering from prenatal anxiety may benefit from support adjusting to the parenting role especially when taking care of an infant with difficult temperament. Future research should consider mechanisms and influential factors pertaining to the relationship between prenatal anxiety and infant temperament.
